# CVD-Grown Monolayer Graphene-Based Geometric Diode for THz Rectennas

**DOI:** 10.3390/nano11081986

**Published:** 2021-08-02

**Authors:** Heng Wang, Gaurav Jayaswal, Geetanjali Deokar, John Stearns, Pedro M. F. J. Costa, Garret Moddel, Atif Shamim

**Affiliations:** 1 IMPACT Lab, Computer, Electrical and Mathematical Sciences and Engineering (CEMSE) Division, King Abdullah University of Science and Technology (KAUST), Thuwal 23955-6900, Saudi Arabia; gaurav.jayaswal@kaust.edu.sa; 2 Physical Science and Engineering Division, King Abdullah University of Science and Technology (KAUST), Thuwal 23955-6900, Saudi Arabia; geetanjali.deokar@kaust.edu.sa (G.D.); Pedro.DaCosta@kaust.edu.sa (P.M.F.J.C.); 3 Department of Electrical, Computer & Energy Engineering, University of Colorado, Boulder, CO 80309-0425, USA; jost3184@colorado.edu (J.S.); moddel@colorado.edu (G.M.)

**Keywords:** THz rectenna, geometric diode, CVD-grown graphene, ballistic transport, Monte Carlo simulation

## Abstract

For THz rectennas, ultra-fast diodes are required. While the metal–insulator–metal (MIM) diode has been investigated in recent years, it suffers from large resistance and capacitance, as well as a low cut-off frequency. Alternatively, a geometric diode can be used, which is more suitable due to its planar structure. However, there is only one report of a THz geometric diode based on a monolayer graphene. It is based on exfoliated graphene, and thus, it is not suitable for mass production. In this work, we demonstrate chemical vapor deposition (CVD)-grown monolayer graphene based geometric diodes, which are mass-producible. The diode’s performance has been studied experimentally by varying the neck widths from 250–50 nm, the latter being the smallest reported neck width for a graphene geometric diode. It was observed that by decreasing the neck widths, the diode parameters such as asymmetry, nonlinearity, zero-bias resistance, and responsivity increased within the range studied. For the 50 nm neck width diode, the asymmetry ratio was 1.40 for an applied voltage ranging from −2 V to 2 V, and the zero-bias responsivity was 0.0628 A/W. The performance of the diode was also verified through particle-in-cell Monte Carlo simulations, which showed that the simulated current-voltage characteristics were consistent with our experimental results.

## 1. Introduction

Researchers are devoting themselves to seek new energy sources which are safe, renewable, and environment-friendly [1,2,3]. Infrared (IR) energy from waste heat in the environment is abundant and a promising candidate as a renewable energy source. The energy from waste heat is within the wavelength range of 2–11 μm, corresponding to frequencies of tens of Terahertz (THz) [4]. Since this IR energy can be considered as very high-frequency electromagnetic waves, they can be captured by a receive antenna and rectified by a diode into direct current (DC). This combination of a receive antenna integrated with a rectifying diode is typically known as a rectenna [5], which is quite common in the microwave frequency range but very challenging to realize in the THz domain.

This approach can provide sustainable energy for 24 h a day without adding any pollution to the environment. However, it requires an extremely small antenna (typically with micro-scale dimensions and nano-scale gaps) and an ultra-high-speed diode (operation frequency at THz or even higher) [6]. Typical semiconductor-based diodes cannot operate at such high frequencies; however, the metal–insulator–metal (MIM) diode could be an attractive option in this scenario [7]. In our previous work, we have demonstrated examples of a zero-bias rectenna system based on MIM diodes [8]. However, MIM diodes have their own set of challenges. The performance of the MIM diode heavily depends on the extremely thin oxide (insulator) layer. Low permittivity of the oxide layer is required to enhance the cut-off frequency to tens of THz, and a very fine oxide layer surface is a must for a smooth and reliable operation of the diode. Furthermore, there are contradicting requirements in terms of oxide thickness. An extremely thin oxide layer (1–3 nm) is required for electrons to tunnel through and for the diode to exhibit a relatively lower resistance; however, a very small thickness causes a large capacitance, which not only negatively effects the cut-off frequency, but also affects the rectification efficiency of the diode. Finally, the properties of the oxide layer vary with the deposition methods and conditions, and thus maintaining reliable and repeatable performance is a challenge [8].

Alternatively, THz diodes can be realized through a completely planar structure, namely, a geometric diode [9]. Compared with the MIM diode, the geometric diode is without any overlap in the vertical direction, so the capacitance is relatively low. However, this diode needs a long mean free path (MFP) material, and thus, in the only previously-demonstrated THz geometric diode, graphene has been used [9]. A monolayer graphene is highly conductive, so the resistance is relatively low as well. These two merits (relatively lower capacitance and resistance) of geometric diodes are attractive for THz rectennas, as they not only ensure THz operation, but also help in matching the antenna impedance. For the geometric diode, the RC constant can be as low as 10^−15^ s, and the 28.3 THz response has been verified [10]. However, the previous graphene-based geometric diode was based on exfoliated graphene [10], which may have better quality than CVD-grown graphene, but it also has drawbacks. First, graphene can be realized in a very small area (typically a small piece of 10–100 μm by 10–100 μm [11]) through the exfoliation process, so this method is not practical for mass manufacturing of devices. Second, the exfoliated graphene is not uniform, so in some regions, it may be multi-layer rather than monolayer. Furthermore, in the previous work, the minimum neck width was restricted to 75 nm, which permitted quasi ballistic operation, keeping in mind that the MPFL of the graphene was around 45 nm in that work.

In this work, CVD-grown monolayer graphene has been used for geometric diode realization, which is an industry-standard process and can be used to fabricate the rectenna systems at a mass scale [12]. The graphene geometric diodes have been fabricated with five different neck widths (including the smallest width of 50 nm, which achieved complete ballistic transport) to study the effect of the neck width on the diode’s performance. This was done by analyzing the asymmetry, nonlinearity, zero-bias resistance, as well as responsivity for the varying neck widths of the diode. It was experimentally verified that the performance of the graphene geometric diode improves as its neck width decreases within the range investigated.

## 2. Materials and Methods

### 2.1. Concept and Working Principle

**Figure 1:**
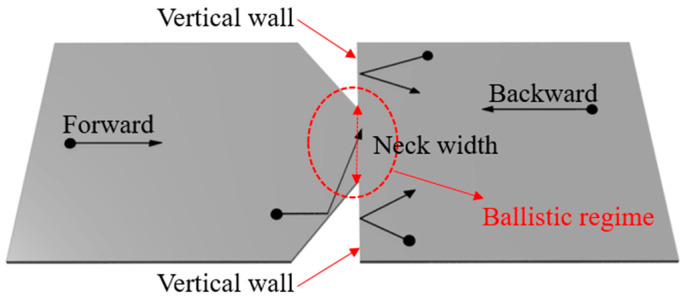
A schematic of the graphene geometric diode.

The design of the geometric diode is shown in Figure 1, and it was realized through monolayer graphene. The left part (forward part) is like a funnel, while the right part (backward part) is like a simple rectangle. Unlike the conventional diodes that rely on either junction or potential barrier, the geometric diode can rectify AC signal to DC based on the asymmetric geometry of the structure [13]. The charge carriers move in the forward direction and pass through the neck width, constituting the forward current (assume that the hole is the majority charge carrier). On the way back, these charge carriers get reflected from the vertical wall, and thus, the backward current is much lower than the forward current. This asymmetry in the forward and backward currents resulted in the diode behavior. To make the movement of the charge carriers in the diode mainly affected by the geometry and not by scattering around the neck regime, we needed quasi-ballistic or ballistic transport in that regime, which meant the neck width of the geometric diode had to be around or less than the MFP of the graphene [14].

### 2.2. Fabrication

**Figure 2:**
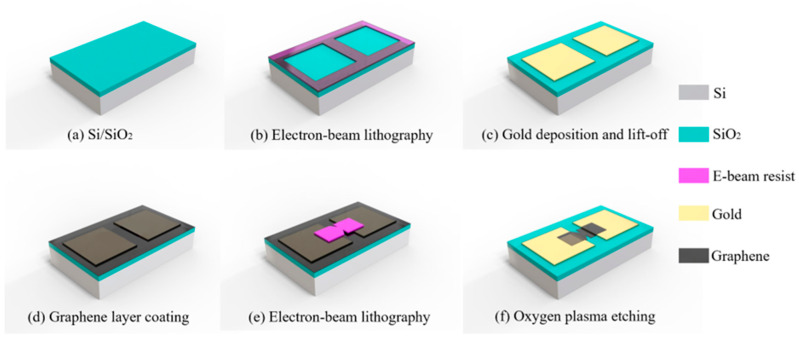
The fabrication process of the graphene geometric diode. (**a**) SiO_2_ deposition on Si by PE-CVD; (**b**) ZEP520A resist spin-coating, EBL exposure and development; (**c**) Gold sputtering and lift-off; (**d**) Monolayer graphene transfer; (**e**) PMMA 950K A4 spin-coating, EBL exposure and development; (**f**) Final shape of the geometric diode after oxygen plasma etching.

**Figure 3:**
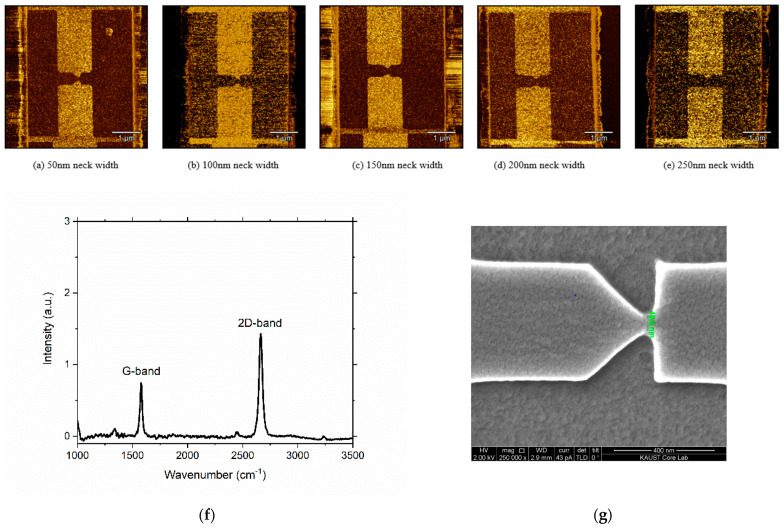
AFM images of the graphene geometric diode (the black area is graphene) with different neck widths. (**a**) 50 nm neck width, (**b**) 100 nm neck width, (**c**) 150 nm neck width, (**d**) 200 nm neck width, (**e**) 250 nm neck width, (**f**) Raman spectrum of the graphene in the gap, (**g**) SEM image of the 50 nm neck width geometric diode.

The fabrication of the geometric diodes was done in the in-house cleanroom through high-resolution e-beam lithography (EBL) processes, as shown in Figure 2. A highly doped Si wafer was used as the substrate, and a 300 nm layer of SiO_2_ was deposited on it by plasma-enhanced (PE)-CVD (Figure 2a). This oxide layer provided insulation as well as helping in the visibility of the monolayer graphene [15]. A layer of e-beam resist ZEP520A was spin-coated on the oxide layer first, and then, it was exposed by EBL. It was developed in the ZED-N50 developer to form two square-shaped trenches (Figure 2b), after which a Ti (10 nm)/Au (40 nm) layer was deposited on the sample through sputtering. Eventually, two metal pads were realized through the lift-off process (Figure 2c). The monolayer graphene, grown by CVD on Cu foil, was then transferred on the sample with Ti/Au electrodes. (Figure 2d) [16,17,18]. In the next step, an e-beam resist PMMA 950K A4 was spin-coated and patterned by EBL, precisely in the gap between the metal pads (Figure 2e). In the final step, oxygen plasma etching was utilized to realize the graphene geometric diode (Figure 2f). The most challenging part of the fabrication was to achieve a 50 nm resolution for the neck width while maintaining the shape of the device. The diode needed to be precisely aligned in the gap between the pads; otherwise, it can completely move to one of the pads and get short-circuited. To verify the fabrication process, AFM (atomic force microscopy) was used. AFM images of the geometric diodes with different neck widths are shown in Figure 3. The increasing neck width can be observed in the AFM images in Figure 3a–e, and the Raman spectra of the graphene in the gap in Figure 3f verified that the graphene was monolayer, as the peak at the G-band versus the peak at 2D-band was 1:2 [19]. To confirm that we achieved a neck width of 50 nm, the SEM image of the geometric diode is shown in Figure 3g, which was obtained before the etching of the graphene (refer to fabrication step shown in Figure 2e).

## 3. Results and Discussion

### 3.1. Electrical Characterization

**Figure 4:**
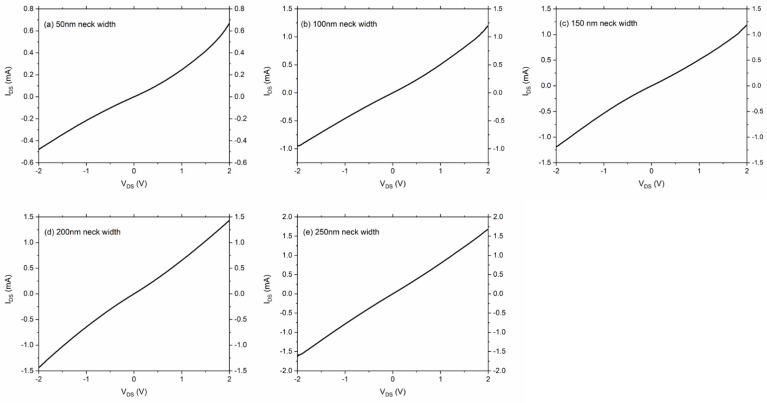
I-V characteristics of the geometric diode with different neck widths under V_GS_ = 0 V. (**a**) 50 nm neck width, (**b**) 100 nm neck width, (**c**) 150 nm neck width, (**d**) 200 nm neck width, (**e**) 250 nm neck width.

The fabricated devices were characterized by a Keithley 4200 SCS analyzer, and I-V (current-voltage) characteristics were measured for the geometric diode with different neck widths varying from 50 nm to 250 nm. The drain-source voltage VDS was swept from −2 V to 2 V, while the back gate voltage was set to VGS = 0 V. Figure 4 shows how the I-V characteristics changed with the increasing neck width.

**Figure 5:**
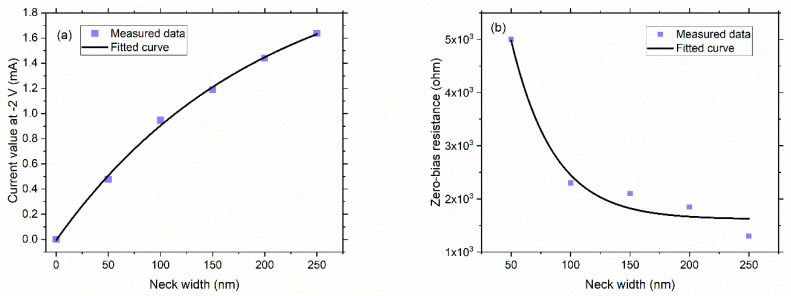

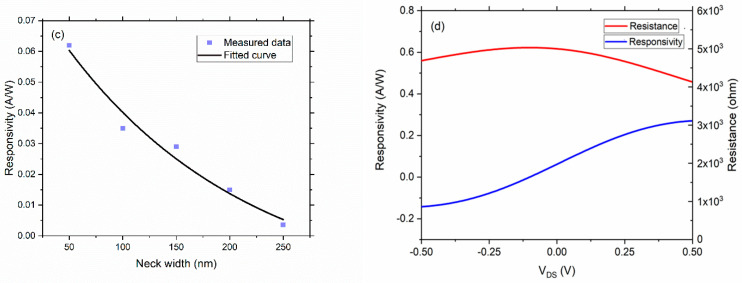
(**a**) Exponential-fitted curve for the magnitude of current at −2 V; (**b**) Exponential-fitted curve for the zero-bias differential resistance of the geometric diode with different neck width; (**c**) Exponential-fitted curve for the zero bias-responsivity of the geometric diode with different neck width. (**d**) The resistance and responsivity of the 50 nm neck width geometric diode at a small V_DS_ range of −0.5 V to 0.5 V.

As can be seen from Figure 4, there were clear trends for the magnitude of the current, nonlinearity, and asymmetry, as the neck width of the geometric diode increases. As expected, the magnitude of the current increased as the neck width increased from 50 nm to 250 nm. This was because, for the same V_DS_, more charge carriers could pass through the neck per second as compared to the case of the smaller neck widths. This increase in the current with respect to the increasing neck widths can be clearly seen in Figure 5a for −2 V of V_DS_. Furthermore, the nonlinearity started to diminish as the neck width increased. This was because the funnel shape started to look more like a rectangle, which made the IV characteristics similar to a resistor. The asymmetry was assessed through an asymmetry ratio, which was defined as the magnitude of the forward current over the magnitude of the backward current under a specific V_DS_, as shown in Equation (1) [20].

(1) $$A={\left|\frac{I_{forward}}{I_{backward}}\right|}_{\left|V_{DS}\right|=constant}$$

At *V_DS_* = 2 V, the asymmetry ratio for the geometric diode with 50 nm and 100 nm neck widths were 1.40 and 1.25, respectively. For the other three neck widths, the asymmetry ratios were almost 1. It is worth mentioning here that the MFP of the graphene was 70 nm, and thus, for the 50 nm neck width, the geometrical shape of the diode had the most effect on the movement of the charge carriers. For the larger neck widths, the neck widths were much greater than the MFP of the graphene; thus, the charge carrier transport around the neck regime could not be considered ballistic anymore [21], and the movement of the charge carrier was mainly affected by scattering instead of the geometrical shape of the diode.

Furthermore, the zero-bias differential resistance and zero-bias responsivity at *V_GS_* = 0 V (here, zero-bias means zero drain-source voltage, so both *V_DS_* and *V_GS_* were zero) of the diode were also extracted for different neck widths. Both zero-bias differential resistance and responsivity tended to decrease with the increasing neck widths, where the measured data has been fitted in Figure 5b,c. These results are consistent with the I-V characteristics reported for a silicon geometric diode [22]. A similar trend for Hall resistance and nonlinearity was also observed in a Hall sensor experiencing the ballistic effect [23]. As has been discussed before, larger neck widths result in lower non-linearity within this range (indicative from the linear I-V curves). Thus, the zero-bias resistance and responsivity were also lower for larger neck widths, because they are directly related to the nonlinearity of the device. An I-V curve with a higher nonlinearity had a smaller slope at zero bias, leading to a large zero-bias resistance. Similarly, responsivity is a measure of the output current produced for a given input power [10], as expressed in Equation (2) [24]. With the diode becoming more linear, the $I^{\prime \prime }\left(V\right)$ on the numerator in Equation (2) will become smaller.

(2) $$\mathrm{Responsivity}=\frac{1}{2}\frac{I^{\prime \prime }\left(V\right)}{I^{\prime }\left(V\right)}$$

For the 50 nm neck width geometric diode, the responsivity at zero bias was calculated to be 0.0628 A/W. For a rectenna system using this geometric diode, it meant that 0.0628 A net current could be obtained with an input power of 1 W if it was perfectly coupled with the antenna. According to author’s best knowledge, this is the highest reported zero-bias responsivity from a graphene geometric diode, which is double that of the previously reported value of 0.0285 A/W (at *V_GS_* = 0 V for a 75 nm neck width geometric diode [9]). Furthermore, to clearly show the nonlinearity of the I-V curve at a small V_DS_, the differential resistance and responsivity of the 50 nm neck width geometric diode at *V_DS_* of −0.5 V to 0.5 V is shown in Figure 5d. It is also worth mentioning that by applying a small bias to the diode, the resistance could be reduced, and the responsivity could be increased to a higher order (more than 0.2 A/W at *V_DS_* = 0.5 V), even though we preferred zero-bias for practical application.

### 3.2. Verification through Monte Carlo Simulation

For verification purposes, the geometric diode current-voltage characteristics were also obtained through a particle-in-cell Monte Carlo simulation method [25]. In this method, individual charge carriers were lumped together into macro-particles and were allowed to move under the influence of electric fields within the device. After a voltage was applied as a boundary condition on the electrodes, the electric field was calculated throughout the device. The electric field perturbed the velocities of the particles, which would otherwise be randomly directed, and the particles hopped forward over a small time step. Each particle periodically had its velocity reset in a random direction at a rate that was determined by the collision time of the diode material, which was related to the MFP length by the Fermi velocity. Particles were reflected specularly off device boundaries and counted as they passed through either electrode. This process continued for a predetermined number of time steps, 10^4^ for the results shown here, and the current was computed from the total amount of charge that passed through the device in the given simulation time.

**Figure 6:**
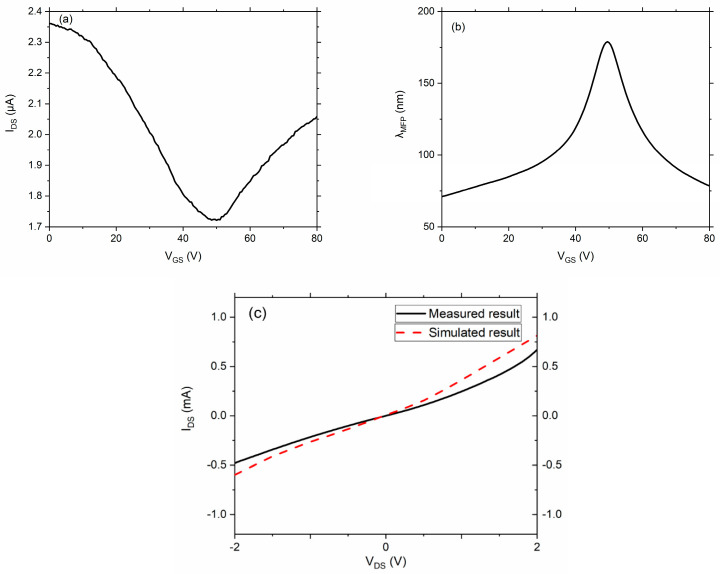
(**a**) Measurement of the gate voltage-dependent drain-source current of a graphene strip on Si/SiO_2_. The strip was 500 nm wide and 3.9 μm long. The current reached a minimum at *V_GS_* = *V_D_* = 50 V. A source-drain voltage of *V_DS_* = 10 mV was used for the measurements; (**b**) Mean-free-path lengths deduced from the graphene sample in Figure 6a. At zero gate voltage, the charge carriers had λ_MFP_ = 70 nm; (**c**) Simulated current-voltage compared with measured current-voltage characteristics of a geometric diode with a 50 nm neck width. The charge carriers were assumed to have λ_MFP_ = 70 nm.

The charge carrier MFP length and the gate voltage applied to the graphene were both required inputs to the simulator. Both were deduced from the gate voltage-dependent current measurements of the graphene before it was patterned into the diode shape. Figure 6a shows these measurements for a graphene strip of length *L* = 3.9 μm and width *W* = 500 nm on a 300 nm SiO_2_ substrate. The SiO_2_ was formed on a highly doped silicon wafer, which served as the gate electrode and was held at voltage *V_GS_* relative to the source terminal of the graphene strip. A source-drain voltage of *V_DS_* = 10 mV was used for these measurements. It is worth mentioning here that the measurements for MFP were done on the same substrate and deposited graphene layer where the diodes were formed.

The Dirac voltage, *V_D_*, is the voltage where minimum conductivity occurs due to minimum free-carrier density. For this sample, it was around 50 V. We used the model of Dorgan et al. to estimate the concentration of free electrons, *n*, and holes, *p*, as a function of gate voltage and to subsequently estimate the graphene mobility [26]. We calculated the mobility as

(3) $$\mu =\frac{L}{W}\frac{I_{DS}}{q\left(n+p\right)V_{DS}}$$

where *q* is the elementary charge and the gate dependence is included in *n* and *p*. The carrier mean-free-path length, *λ_MFP_*, is then computed as [13]

(4) $$\lambda_{MFP}=\frac{h}{2q}\mu \sqrt{\frac{n+p}{\pi }}$$

The result is shown in Figure 6b, which gave *λ_MFP_* = 70 nm at *V_GS_* = 0, or 50 V from the Dirac voltage. A maximum mean free path length could be obtained when the gate voltage V_GS_ was around 50 V, but we still used zero V_GS_, because we aimed to build a zero-bias rectenna (an energy harvesting device which does not require any additional bias for its own operation), which meant we would not provide any bias to it (both zero *V_DS_* and zero *V_GS_*) for practical application.

The simulator was run for a graphene sample with *λ_MFP_* = 70 nm, with a gate voltage of 0 V. *V_D_* was used in the simulator to model the free carrier concentration as well as the effective mass, both of which depend on *V_GS_–V_D_* [27]. Figure 6c shows the simulated current-voltage curve along with the measured current-voltage curve for a geometric diode with a neck width of 50 nm and electrode width of 500 nm. The simulated curve had a similar trend as that of the measured curve in general; however, the simulated current was slightly larger than the measured current. According to our analysis, the difference between simulations and measurements came from the variance in the values extracted from the measured Dirac curve. These values were used in the simulator, so from the simulation’s perspective, two aspects are important to discuss. (1) We used the formula $n=C\left|V_{GS}-V_{Dirac}\right|/q$ as in reference [26] to calculate the charge concentration, n, where C is the capacitance per unit area for the 300 nm thick SiO_2_. However, the deposited SiO_2_ was not exactly 300 nm, which affected the value of C and accuracy of the charge concentration n. This further affected the mean free path length that we used in the simulator. (2) The quality of the graphene also varied slightly in the sample, so the mean free path length measured from the graphene strip could also be slightly different for the 50 nm neck width device. Nonetheless, the simulator can predict the behavior of the device within a decent level of accuracy.

## 4. Conclusions

Geometric diodes with five different neck widths were fabricated utilizing a CVD-grown monolayer graphene. Compared to the reported 75 nm neck width geometric diode based on exfoliated graphene, the proposed diode in this work had a neck width of 50 nm, which ensured completely ballistic transport, as the MFP of the graphene was 70 nm at zero V_GS_. The zero-bias responsivity was calculated to be 0.0628 A/W, which was much higher than the previously reported value of 0.0285 A/W, meaning much higher output of the rectenna could be achieved using this geometric diode. The asymmetry ratio of the proposed geometric diode could be up to 1.40. CVD-grown graphene greatly simplifies the fabrication process and is suitable for mass production of such devices. We experimentally studied the nonlinearity, asymmetry, zero-bias resistance, and zero-bias responsivity of the diode, where all parameters increased if the neck width decreased. It can thus be concluded that the smaller the neck width, the better the performance of the geometric diode within the range investigated. For verification purposes, simulations were performed for the 50 nm neck width diode through the particle-in-cell Monte Carlo method, which demonstrated a performance similar to our experimental results.

## Data Availability

The data presented in this study are available on request from the corresponding author.
